# An Overview on Methods, Evidence, and Study Quality of Health Economic Evaluation Studies for Independently Usable Digital Health Apps: Rapid Review

**DOI:** 10.2196/68349

**Published:** 2025-08-19

**Authors:** Valerie Anne Alber, Hendrikje Rödiger, Alessandro Campione, Juliane Schiller, Dominik Burziwoda, Cornelia Henschke

**Affiliations:** 1 Perfood GmbH, Lübeck, Germany; 2 Department of Health Care Management, Faculty of Economics and Management, Technische Universität Berlin, Straße des 17. Juni 135, Berlin, 10623, Germany, 49 30 31428732; 3 Private Institute for Applied Health Service Research (inav), Berlin, Germany; 4 Institute of General Practice and Interprofessional Care, University Hospital Tübingen, Tübingen, Germany

**Keywords:** digital health application, DiHA, DiGA, digital health, digital health intervention, cost-effectiveness, health economics, economic evidence, cost-effective, efficacy, rapid review

## Abstract

**Background:**

While research on the efficacy of digital health applications (DiHA) is progressing, health economic evaluations (EEs) remain limited but are urgently needed to guide reimbursement and coverage decisions. Existing health policy frameworks frequently overlook cost-effectiveness considerations, and many studies presuppose cost savings without sufficient empirical validation. Although previous reviews have assessed digital health interventions more broadly, none has specifically focused on the cost-effectiveness of those intended for independent patient use.

**Objective:**

This rapid review aims to summarize the current economic evidence and the methods used in health EEs, including modeling practices, and assess the quality of health economic studies on independently usable DiHA for patients.

**Methods:**

A systematic search was conducted in 4 electronic databases (PubMed, Cochrane Library, EconBiz, and Web of Science), supplemented by both systematic and unsystematic hand searches. Studies were included on predefined inclusion criteria, considering only complete health EEs of DiHA intended for independent patient use. Data were narratively synthesized. Risk of bias (RoB) was assessed using the Cochrane risk of bias tool 2 (RoB 2), and methodological quality was evaluated using the Consensus on Health Economic Criteria (CHEC) checklist and the Consolidated Health Economic Evaluation Reporting Standards (CHEERS). The review adhered to PRISMA (Preferred Reporting Items for Systematic Reviews and Meta-Analyses) guidelines for implementation and reporting.

**Results:**

A total of 3841 results were identified. After screening the full texts of 82 publications, 7 studies were included in the final analysis. Four of the studies concluded that the app under review was cost-effective compared to the chosen control group. Most of the studies that provided economic evidence incorporated indirect costs and used a societal perspective. All studies used cost-utility analyses (n=7), with the majority based on randomized controlled trials (RCTs) (n=5), considering the health care payer perspective (n=3). Standard care was the most common comparator (n=5). Health outcomes were primarily measured using the EQ-5D (n=3) and condition-specific instruments (n=7). The incremental cost-effectiveness ratio, reported as costs per quality-adjusted life year, was the most frequently reported outcome (n=4). Overall, the quality of the EEs was rated positively using the CHEERS 2022 and CHEC checklists. However, more than half of the underlying RCTs exhibited a high RoB.

**Conclusions:**

DiHA have the potential to be cost-effective, and evaluations of these are of increasing interest. However, health EE is not yet routinely applied in their assessment. Improved reporting of RCT outcomes and greater consistency in modeling practices are needed to support robust EEs in this domain, which could advance evidence-based decision-making and reimbursement policies. This review focused on studies of indication-specific apps, which may have excluded broader applications, highlighting opportunities for more comprehensive research as the field evolves.

## Introduction

Digital health applications (DiHA) play an increasingly important role in a rapidly evolving society. This is especially relevant in light of the growing burden from diseases, such as diabetes, cancer, cardiovascular disease, and mental illnesses [1]. Digital health solutions offer promising approaches to effectively address challenges of these diseases by enabling patients to adapt their lifestyle or strengthen their ability to cope with a disease. The scalability and universal accessibility of DiHA hold great promise for alleviating the strain on health care systems [2-4]. Despite this potential, there is no internationally standardized definition for the term “digital health applications (apps)” [5]. In this study, we only consider indication-specific digital health apps that can be used by patients without service providers such as doctors or therapists and without further sensors, devices, and wearables that go beyond the smartphone.

Despite the opportunities, health systems are still in the early stages of developing and implementing approaches to identify safe and effective DiHA for determining coverage eligibility and pricing [5]. Pioneering countries such as Germany, France, and Belgium have developed their own approach for the assessment and reimbursement of DiHA [5,6]. However, there is no standardized approach for evaluating the clinical and economic outcomes of DiHA [7,8]. Health economic evaluations (EEs) that are required for determining the coverage and pricing of technologies such as pharmaceuticals and medical devices have often been ignored in planned or already implemented policy approaches [9]. Due to the novelty of the area of DiHA, evidence regarding cost-effectiveness is scarce. Appropriate cost-effectiveness considerations can play an important role in informing decisions about the introduction of digital health technologies into health care systems [10]. Some countries have already started to include DiHA in their health benefit baskets. As health care systems face growing cost pressures, it is essential to consider the health economic dimension of these applications. However, health EE is not solely about reducing expenditures; rather, it focuses on determining whether the costs and benefits of digital health interventions are appropriately balanced, typically assessed through comprehensive cost-effectiveness or cost-utility analyses (CUAs).

The benefit of technologies can also be assessed in a multidisciplinary and systematic process known as health technology assessment (HTA). By assessing clinical effectiveness, safety and costs, and other relevant domains, HTA provides information for decision-making to promote efficient and high-quality health care systems [11]. The use of HTA for evaluating DiHA is not yet common practice. However, as some DiHAs are classified as medical devices and are increasingly being integrated into standard care, it remains to be seen whether this will change in the future.

In some publications that analyze the effectiveness of DiHA in health care, cost-effectiveness is simply assumed at the outset across several indication areas without any source or evidence being provided [12]. In a study examining mHealth (medical health interventions that can be used through mobile devices such as smartphones, patient monitoring devices, personal digital assistants, and other wireless devices [13]) evaluations from 12 HTA organizations, it was found that not a single published EE was considered, but that all were assumed to be cost-saving [14]. An analysis of DiHA frameworks by the Austrian Institute for Health Technology Assessment GmbH [15] found that 4 out of 6 frameworks mentioned economic aspects as an assessment domain. Nevertheless, cost-effectiveness per se was explicitly considered in only 1 framework. Although a recently published systematic literature review showed an increasing body of evidence on cost-effectiveness analyses (CEAs) of digital health interventions, the body of evidence on cost-effectiveness of DiHA remained limited. Of the 35 CEAs included, only 3 focused on mobile phone-based systems and applications, while most were related to video conferencing systems [10].

Overall, EEs of digital health interventions, including DiHA, are a challenge, as they are not well understood and are still at an early stage of development and implementation from a methodological perspective. Key distinct features of digital health interventions must be considered in EE. Those include, for instance, active user input from patients or physicians that is necessary for proper use as intended. Digital health interventions can lead to changes in health outcomes as well as structural and procedural changes in healthcare. But impacts may also include changes outside of the health care sector. Therefore, cost per quality-adjusted life year (QALY) or an incremental cost-effectiveness ratio (ICER) alone may be inappropriate to reflect the broad range of impacts. A combination with a cost-consequence analysis is more suitable and should be applied [16]. In addition, the use of an impact matrix is recommended to list potential multidimensional effects of digital health interventions on various stakeholders [17]. Moreover, the rapidly evolving nature of digital health interventions at the design stage, as well as a broader perspective beyond that of national health services or insurers, should be considered for EE [16]. Previous reviews on the application of health EE have focused on digital health interventions either in general or within specific therapeutic areas. However, no review has been identified that examined the cost-effectiveness of DiHA that can be used independently by patients without the need for additional sensors or devices.

The aim of this study is to identify full health EEs of apps that can be used independently, without the involvement of service providers, additional devices, or external devices such as wearables. This approach ensures that the analysis focuses solely on the cost-effectiveness of the apps themselves, excluding potential confounding effects from other service components. Specifically, the study seeks to (1) provide an overview of current practices in health EE, (2) summarize the existing economic evidence, and (3) assess the methodological quality and RoB of the included studies.

## Methods

### Study Process

We used the rapid evidence review approach, as it provides a streamlined alternative to standard systematic reviews. In conducting this review, we adhered to the general methodological guidance provided by the Cochrane Rapid Reviews Methods Group [18,19]. For reporting, we followed the Preferred Reporting Items for Systematic Reviews and Meta-Analyses (PRISMA) 2020 statement, along with the guidance for systematic reviews without meta-analysis [20,21] (completed checklist in Multimedia Appendix 1).

### Eligibility Criteria

The inclusion and exclusion criteria were defined by using the PICOS (Population, Intervention, Comparison, Outcomes, and Study type) framework [18] Publications were included if the population was patients using digital applications. The intervention includes DiHA that are used by patients independently. There was no restriction regarding the comparison group as long as one was present. The study type is limited to trial-based or model-based full health economic studies. Publications were included if they were published between 2008 and 2023, as apps came onto the market from this period onwards. Table 1 shows the inclusion and exclusion criteria of the study.

**Table 1.** Inclusion and exclusion criteria.

	Inclusion criteria	Exclusion criteria
Population (P)	Patients using digital health applications	Service providers or health care professionals using digital health applications
Intervention (I)	Digital health applications that can be used independently by patients. The digital health application is the only component of the intervention and requires no further support during its use. Except for an introduction to the application, no further point of action is required by the service provider.	Interventions that do not correspond to the definition of digital health applications included. Those include interventions that act as communication technology between patients and a service provider are designed as web applications are apps that do not exclusively serve a health purpose. Digital health applications that are part of an intervention such as a health care program. Interventions that require additional wearables, sensors, or devices that go beyond the functions of the digital health application.
Comparison (C)	Any kind of comparator group	No comparator
Outcome (O)	Consequences (eg effectiveness, utility, benefit) and costs	Consequences only
Study type (S)	Trial-based and model-based, full health economic evaluations: Cost-effectiveness analysis (CEA) Cost-minimization analysis^a^ (CMA) Cost-utility analysis (CUA) Cost-benefit analysis (CBA)	Case studies, commentaries, letters, cost-of-illness studies, non-comparative cost studies, protocols, reviews, systematic reviews
Filter	Period: 2008 until April 2023	

a Cost-minimization analysis is considered as it represents a form of comparative economic analysis that compares the costs of alternatives that are assumed to have equivalent consequences.

### Information Sources

#### Systematic Literature Search in Electronic Databases

Databases from the subject areas of medicine (PubMed and Cochrane Library), economics (EconBiz), and a multidisciplinary perspective (Web of Science) were searched to capture all relevant subject areas. By selecting several databases, the retrieval bias was kept to a minimum [22]. The search strategy used was reviewed using the Checklist for the Peer Review of Electronic Search Strategies [17].

#### Hand Search

In addition to database searches, a free web search, an unstructured search using publicly available web-based resources, such as search engines or open-access websites, was carried out using the search terms from the systematic literature search. Further literature retrieval methods included forward and backward searches [23] in the bibliographies and “cited by” information from reviews that, for example, examined health economic studies of mHealth interventions (eg, [24,25]). Furthermore, as recommended by Guba [26], the HTA database of the Centre for Reviews and Dissemination York (Centre for Reviews and Dissemination 2022) was reviewed. Additionally, a hand search of health economic studies of the DiHA included in the German DiHA directory, an official registry by the Federal Institute for Drugs and Medical Devices, listing approved DiHA that are reimbursed by statutory health insurance in Germany, was carried out. For this purpose, the names of all apps listed in the DiGA directory in combination with cost-effectiveness terms were entered into Google Scholar.

### Search Strategy

The database search strategies consisted of search terms related to the intervention and study type. The search was divided into 2 blocks: The first block contained synonyms and generic terms for DiHA. The second block contained terms for health economic study types. The blocks were strung together using Boolean operators (AND and OR), and the respective syntax of the databases was applied. The detailed search strategies can be found in Multimedia Appendix 2.

### Selection Process

To minimize the risk of publications being incorrectly included or excluded and to reduce selection bias, 2 reviewers (VA, JS) independently screened a random 20% sample of all records based on their titles and abstracts, while 1 reviewer (VA) screened the entire dataset [18]. Any discrepancies were resolved through consensus. The principle of (at least) 2 screeners is common practice and is recommended by the Agency for Healthcare Research and Quality, the Center for Reviews and Dissemination, the Institute of Medicine, and the Cochrane Collaboration to ensure proper application and formulation of the inclusion and exclusion criteria, as well as to prevent errors [27]. Disagreements were resolved through discussion until consensus was reached. As agreement between both reviewers was sufficiently high (above 80% raw agreement), the remaining records were screened by 1 reviewer (VA). After retrieving the full-text articles for all potentially eligible studies, each full-text article was screened for eligibility by 1 reviewer (VA). A second reviewer (JS) then screened all full-text articles excluded by the first reviewer (VA). Consensus of disagreements was again reached by discussion and by consulting additional reviewers (CH, HR). Records retrieved from databases were organized and managed using the online version of Rayyan Systems, Inc.

### Data Collection Process

Data on general characteristics, methods, and outcomes of the included. EEs were extracted into a standardized Excel sheet (VA), which was piloted by 2 other reviewers (CH, HR). All extracted data were verified by the 2 additional reviewers (CH, HR). Disagreements were resolved by consensus.

### Risk of Bias and Quality Assessment of Included Economic Evaluation Studies

To evaluate the quality of the health economic studies, 3 different assessment tools were used. These tools differ in their scope, criteria, and evaluation methods [28], and their combined use allows for a more comprehensive assessment by covering both clinical validity and economic robustness. The Cochrane risk of bias tool 2 (RoB 2) considers biases that arise at different stages of a trial. The Consolidated Health Economic Evaluation Reporting Standards 2022 (CHEERS 2022) assess the reporting quality of EEs, while the Consensus Health Economic Criteria assess the methodological quality of trial-based EEs. For model-based EEs, the CHEC was extended with an additional item addressing model assumptions and validation. The following assessments were conducted independently by 2 authors.

#### Risk of Bias Assessment (Cochrane Risk of Bias Tool 2 [RoB 2])

To document potential flaws in the evidence summarized and contribute to the certainty in the overall evidence, the risk of bias (RoB) in each study of randomized controlled trial (RCT)-based EEs was assessed using the RoB 2 tool. The RoB 2 tool is specific to a single trial result, ie, an estimate of the relative effect of 2 interventions on a particular outcome. It considers biases that arise at different stages of a trial (ie, bias domains) [29]. RoB 2 was applied independently by 2 authors (AC, HR) to assess the RoB in each study. The robvis visualization tool, a web-based application designed to create visualizations of risk-of-bias assessments, was used to illustrate the RoB for each outcome domain separately [30].

#### Assessment of Publication Bias (Consolidated Health Economic Evaluation Reporting Standards [CHEERS] Statement)

We used the CHEERS 2022, a 28-item checklist, to assess the reporting quality of EEs. The checklist is intended to ensure accurate reporting of which health interventions are compared, in what context, how the evaluation was conducted, what the findings include, and other details [31]. The CHEERS checklist was applied independently by 2 authors (HR, VA) for each study.

#### Assessment of Methodological Quality (Consensus on Health Economic Criteria [CHEC] Checklist)

The methodological quality of trial-based EEs was assessed using the Consensus Health Economic Criteria (CHEC) extended checklist. The CHEC list, designed for conducting systematic reviews based on EEs studies [32], was extended with a question on model assumptions and validation for model-based EEs [33]. The CHEC list was applied for methodological quality assessment of each study by 2 authors (CH, AC) independently. It includes 19 or 20 criteria to be answered with “yes” or “no” depending on whether or not sufficient attention is given to specific aspects, or “not applicable” [32].

### Synthesis Methods

A structured narrative synthesis was performed to synthesize the evidence in a transparent and reproducible manner. Studies were described, grouped by themes, compared, and assessed for evidence strength. EEs were differentiated by design (model-based vs trials-based) and analysis type (CEA, cost-minimization analysis, CUA, cost-benefit analysis).

## Results

### Study Selection

**Figure 1.**
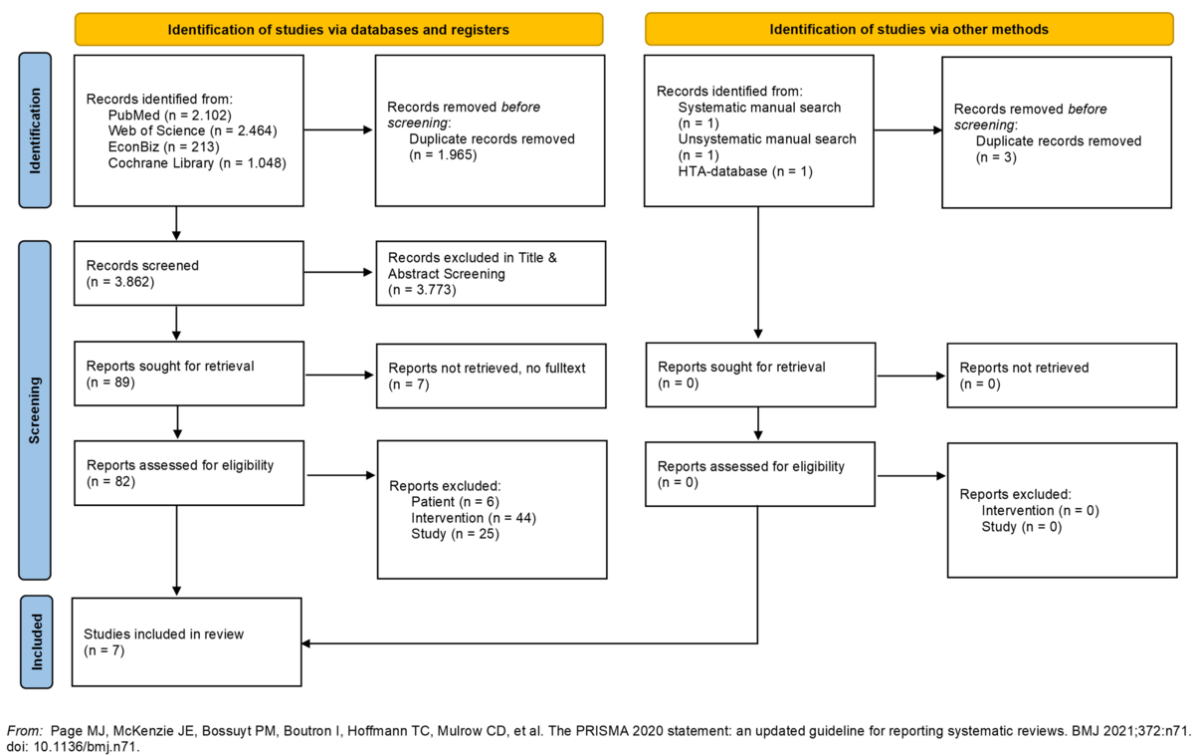
PRISMA (Preferred Reporting Items for Systematic Reviews and Meta-Analyses) flowchart.

The initial search in the PubMed, Web of Science, and EconBiz databases was conducted in April 2022. The search strategy of the Cochrane Library was implemented in May 2022. An update and thus a new search was carried out in the same databases in April 2023. The study selection process is presented in Figure 1. In total, 7 studies were included [34-40]. A list of articles excluded during full-text screening, along with the reasons for their exclusion, can be found in Multimedia Appendix 3.

### Characteristics of Economic Evaluations

The characteristics of the 7 EEs included are shown in Table 2. The study setting was Europe for 6 out of 7 studies, and Asia for 1 study [40]. The indications in the studies range from primary prevention to treatment. The medical specialties differ and range from urology [34,36,39], geriatrics [35], orthopedics [37], psychotherapy [38] to gynecology [40], although half of the apps can be located in the field of gynecology [34,36,39,40]. Accordingly, the active components of the apps vary widely as those are indication specific. Nevertheless, frequent indication of specific functions of the apps includes delivering medical information, sending reminders, and maintaining diary functions. The most common control group in the studies included was standard care.

**Table 2.** General characteristics of included studies.

Author (year of publication)	Setting (country)	Registration no. (study acronym)	Study perspective	Population	Population demographics (sex, age)	Intervention (active components of the app)	Control	Sample size (total, IG; CG)
Ekersund et al (2022) [34][Bibr R35]	Sweden	NCT03097549 (Tät II)	Society	Women with mixed urinary incontinence and urgency urinary incontinence	Female: 100% (IG^a^ and CG^b^); mean age (SD): 58.9 (9.2) (IG), 57.6 (9.9) (CG)	11-step pelvic floor muscle training, 7-step bladder training, medical information and psychoeducation	App with information, but without treatment program	n=122 (IG =60, CG =62)
Ghani et al (2022) [Bibr R36][35]	Sweden	NCT03325699 (SMART4MD)	Health care provider	People with mild cognitive impairment (PwMCI), mild dementia	Female: 44% (IG), 40% (CG); mean age (SD): 76.13 (5.06) (IG), 76,31 (5.18) (CG)	Reminders (medication, doctor appointments, etc.) and cognitive support (cognitive-stimulating games, photos) along with standard care	Standard care	n=345 (IG =173, CG =172)
Loohuis et al (2022) [Bibr R37][36]	The Netherlands	Trial NL4948 (URinControl)	Society	Women with stress, urge, or mixed incontinence	Female: 100% (IG and CG); mean age: 54.9 (SD 12.5) (IG), 52 (SD 9.8) (CG)	Pelvic floor muscle training, bladder training, memories	Care-as-usual	n=262 (IG =131, CG =131)
Pelle et al (2022) [Bibr R38][37]	The Netherlands	NTR6693/ NL6505 (Dr. Bart)	Payer	People with osteoarthritis of the knee and hip	Female: 68.7% (IG); 74.7% (CG); mean age: 62.1 (SD 7.7) (IG), 62.1 (SD 7.0) (CG)	Targets and rewards for healthier lifestyles, reminders	Care-as-usual	n=427 (IG =214, CG =213)
Röhr et al (2021) [Bibr R39][38]	Germany	DRKS00013782 (Sanadak)	Payer	Syrian refugees with mild to moderate post-traumatic stress disorder	Female: 33.8% (IG), 42.6% (CG); mean age: 32.98 (SD 11.0) (IG), 33.67 (SD 11.4) (CG)	Psychoeducation, medical information, videos and audios	Psychoeducational reading material, identical to app information	n=133 (IG =65, CG =68)
Sjöström et al (2017) [Bibr R40][39]	Sweden	NCT01848938 (Tät)	Society	Women with stress incontinence	Female: 100% (IG and CG); mean age: 44.8 (SD 9.7) (IG), 44.7 (SD 9.1) (CG)	Pelvic floor muscle training, medical information	Standard care	n=123 (IG =62, CG =61)
Song and Kanaoka (2018) [Bibr R41][40]	Japan	UMIN000025513 (Karada-no-kimochi)	Health insurance and companies	Working women aged 20‐45	Female: 100% (IG and CG); mean age: 33.0 (IG), 33.0 (CG)	Menstrual cycle predictions based on recorded data (menstrual dates, basal body temperature, mental and physical disorders, information and recommendations tracking, information (preventing depression and dysmenorrhea through menstrual management)	No intervention	n=1,526 (IG =612, CG =914)

a IG: intervention group.

b CG: control group.

Sample size ranged from 122 to 1526 patients. Almost all included evaluations are exclusively study-based, and all of them are based on a randomized controlled trial. Two of the included evaluations belong to the hybrid form and are study-based as well as model-based. In total, 3 EEs were performed from a societal perspective, 3 EEs from a payer perspective, and 1 EE from a health care provider perspective.

Only one of the studies was funded by industry [40]. Conflicts of interest were disclosed in 2 publications [34,40]. Information on funding and conflicts of interest can be found in Multimedia Appendix 4.

### Methods of Economic Evaluations

In summary, 7 study-based EEs were included, 2 of which incorporated a model-based component, corresponding to 7 CUAs and 3 CEAs. The time horizons considered in the study-based EEs are linked to the collection dates of the studies and range from 4 months [38] to 1 year [34,36]. Most EEs used a time horizon for effects and costs of 3 months or less. The hybrid studies used time horizons ranging from 3 months to 1 year (extrapolated from a 3 month study). One EE provided information on discounted rates. Multivariate sensitivity analysis was the most common method for calculating uncertainty (Table 3).

**Table 3.** Methods of economic evaluations.

Author (year of publication)	Type of EE, analytic approach	Time horizon	Discount rate	Uncertainty	Handling of missing data
Ekersund et al (2022) [Bibr R35][34]	CUA^a^, study-based (RCT^b^)	3 months, 12 months	Not applicable	Univariate and multivariate sensitivity analysis	After 3 months, 2 participants (3.3%) were lost from the IG^c^, none from the CG^d^. Of the participants in the IG, 85% (51 out of 60) responded to the 1-year follow-up.
Ghani et al (2022) [Bibr R36][35]	CEA^e^ and CUA, study-based (RCT)	6 months	Not applicable	Multivariate sensitivity analysis, bootstrap	Missing health outcome values at 6 months were treated as missing in base case statistical analysis. Missing health outcome data: Sensitivity analysis where participants lost to follow-up were removed from all analyses. Multiple imputations for missing QALYs^f^.
Loohuis et al (2022) [Bibr R37][36]	CEA and CUA, study-based (RCT)	12 months	Not applicable	Sensitivity analysis, bootstrap	No imputation, as the group with follow-up data was representative. One baseline assessment in the group with follow-up data was missed, which led to missing data on the outcome parameters for 1/172 individuals.
Pelle et al (2022) [Bibr R38][37]	CEA and CUA, study-based (RCT)	3 months, 6 months	Not applicable	Multivariate sensitivity analysis, bootstrap	Multiple imputations for missing data; missing data were managed according to the recommendations of the specific questionnaire.
Röhr et al (2021) [Bibr R39][38]	CUA, study-based (RCT)	4 months	Not applicable	Sensitivity analysis	Multiple imputation of missing baseline data using the algorithm of chained equations (StataCorp LLC) with all sociodemographic variables and baseline assessments of outcome variables as predictors.
Sjöström et al (2017) [Bibr R40][39]	CUA, primarily study-based (RCT)/model-based (extrapolation)	12 months, data from 3-months study extrapolated	Not applicable	One-way and multiway sensitivity analyses	NR^g^
Song and Kanaoka (2018) [Bibr R41][40]	CUA, model-based (event-oriented simulation) and partly study-based (RCT)	End of age 45 [RCT 3-months (baseline, months 1,2,3)]	2% per year, cost and effectiveness	Univariate sensitivity analysis	NR

a CUA: cost-utility analysis.

b RCT: randomized controlled trial.

c IG: Intervention group.

d CG: Control group.

e CEA: cost-effectiveness analysis.

f QALY: quality-adjusted life year.

g NR: not reported.

### Results of Economic Evaluations

Table 4 provides an overview of the results from the EEs. More than half of all studies used a form of the EQ-5D questionnaire, among others, to measure the benefits [35-38]. Five of the studies used validated indication-specific questionnaires. In the study by Song and Kanaoka [40], the effect was measured through modeling (event-driven simulation). In 4 of the studies, direct medical, direct non-medical, and indirect costs were included [34,36,39,40] and in 3 of the studies, the costs considered comprised only direct medical costs [35,37,38].

For the cost-benefit ratio, 5 studies used the ICER in cost per QALY as the model output [34-36,39,40]. Pelle et al [37] and Röhr et al [38], among others, used cost-effectiveness acceptability curves to summarize the impact of uncertainty on the result of their EEs.

Overall, in more than half of the studies, the authors concluded that the app under investigation is cost-effective [34,36,39,40]. The authors of 2 of the studies concluded that the apps analyzed were not or only very unlikely cost-effective [37,38], and the authors of 1 publication concluded that the cost-effectiveness was inconclusive [35]. The additional outcomes and costs that were collected are listed in Multimedia Appendix 5.

**Table 4.** Outcomes of economic evaluations.

	Effects				Total costs			Cost-effectiveness	
Author (year of publication)	Outcome measurement	Intervention group results	Control group results	Effect difference (IG^a^ vs CG^b^) (incremental)	Intervention group (absolute, mean (SE^c^))	Control group (absolute, mean (SE))	Cost difference (IG vs CG) (incremental)	ICER^d^ as reported	Uncertainty
Ekersund et al (2022) [35]	QALY^e^, calculated based on ICIQ-LUTSqol^f^, indication-specific	0.0152 (ICIQ-LUTSqol reduction: 7.7, 95% CI^g^ 6.1-9.0)	0.0037 (ICIQ-LUTSqol reduction: 1.7, 95% CI 0.4-3.0)	0,0115	€ 741.62	€ 605.82	€ 135.80	ICER: € 11,770.52	Range: -€9,303.78 to €22,307.67
Ghani et al (2022)^h^ [36]	QALY measured by EQ-5D-3L^i^	0.869 (0.099)	0.876 (0.101)	0.004 (95% CI: −0.009 to 0.002)	€ 8,188 (762)	€ 8,175 (751)	€ 12 (95% CI: −2090 to 2115)	ICER: dominated NMB^j^: € −187	CEAC: e.g. probability that the measure is cost-effective less than 50%. Given a WTP^k^ of € 48,876 per QALY, probability of being cost-effective <50%, CE plane 47% in North-West quadrant (more expensive and less effective)
	Adjusted QoL-AD^l^, indication specific	39.83 (4.66)	39.71 (5.13)	0.3322 (95% CI: −0.42 to 1.08)	€ 8,188 (762)	€ 8,175 (751)	€ 12 (95% CI: −2090 to 2115)	36	
	MMSE^m^ adjusted	27.69 (2.18)	27.42 (2.46)	0.2100 (95% CI: −0.12 to 0.54)	€ 8,188 (762)	€ 8,175 (751)	€ 12 (95% CI: −2090 to 2115)	57	
Loohuis et al (2022) [37]	IIALY^n^ derived from the ICIQ-UI-SF^o^ symptom score, indication specific	0.71± (SD 0.215)	0.66 (SD 0.250)	0.043	€ 1520 (SD 3425)	1680 (SD 3357)	€ −161 (95% CI: −180 to −151)	ICER: € −3696 (95% CI: −6716 to 12 712)	ICER in cost per IIALY and ICER in cost per QALY: cost-effectiveness plane. Bootstrap simulation with 5000 replications showed that 65.6% of the replications indicated lower costs for the app-based treatment
	QALY measured by EQ-5D-5L^p^	0.89 (SD 0.165)	0.91 (SD 0.145)	−0.025	€ 1520 (SD 3425)	€ 1680 (SD 3357)	€ −161 (95% CI: −180 to −151)	ICER: €6379 (95% CI:−4128 to 12769)	
Pelle et al (2022) [38]	QALY obtained with EQ-5D-3L, score 0.0‐0.5	0.36 (0.07)	0.36 (0.07)	0.00 (95% CI: −0.00 to 0.01)	€ 462 (SD 80)	€ 503 (SD 79)	€ −22 (95% CI: −36 to −3)	ICER: Dominating	CEAC^q^: eg, 0.71 probability of cost-effectiveness at a WTP of €10,000 per QALY gained. iNMB^r^ €53 (95% CI: 11 to 94) at a WTP threshold of €10,000 (0.71 probability) and €274 (95% CI: 25 to 573) at a WTP threshold of €80,000 (0.67 probability)
	QALY VAS^s^ obtained with EQ-5D-3L score 0.0‐0.5	0.42 (0.05)	0.42 (0.04)	0.00 (95% CI: −0.00 to 0.00)	€ 462 (SD 80)	€ 503 (SD 79)	€ −22 (95% CI: −36 to −3)	ICER: Dominating	CEAC and iNMB €29 (95% CI: −2 to 60 at a WTP threshold of €10,000 (0.67 probability)
Röhr et al (2021) [39]	QALY, linear interpolation of EQ-5D-5L index scores from baseline to follow-up	0.290 (0.004)	0.294 (0.004)	−0.004 (0.005) (p=0.35)	€ 384 (67)	€ 484 (111)	€ −100 (112) (p=0.35)	NR^t^	Probability of cost-effectiveness of 0.81 for a WTP of 0 € per additional QALY, and 0.2 for a WTP of 50,000 €.
Sjöström et al (2017) [40]	QALY based on ICIQ-LUTSqol, indication-specific	0.01006 (ICIQ-LUTSqo: 34.1 (6.1))	0.00158 (ICIQ-LUTSqol: 34.8 (6.1))	0.00849 (p<0.001)	€ 547.0	€ 482.4	€ 64.6	ICER: € 7,615.5	No explicit CEAC or related probability values for WTP
Song and Kanaoka (2018) [41]	QALY based on QoL^u^ scores of previous literature, indication specific	6.84	6.77	NR	JPY 704,000	JPY 838,000	JPY −134,000	As intervention dominant, ICER was not calculated	No explicit CEAC or related probability values for WTP

a IG: intervention group.

b CG: control group.

c SE: standard deviation.

d ICER: incremental cost-effectiveness ratio.

e QALY: quality-adjusted life year.

f ICIQ- LUTSqol: International Consultation on Incontinence Modular Questionnaire on Lower Urinary Tract Symptoms and Quality of Life.

g CI: confidence interval.

h Only the results for patients are shown.

i EQ-5D-3L: EuroQoL 5-Dimension 3-Level.

j NMB: net-monetary benefit.

k WTP: willingness-to-pay.

l QoL-AD: Quality of Life Alzheimer Disease.

m MMSE: Min-Mental State score.

n IIALY: incontinence impact adjusted life years.

o ICIQ-UI-SF: International Questionnaire on Incontinence - Short Form Urinary Incontinence.

p EQ-5D-5L: EuroQoL 5-Dimension 5-Level.

q CEAC: cost-effectiveness acceptability curve.

r iNMB: incremental net-monetary benefit.

s VAS: Visual Analog Scale.

t NR: not reported.

u QoL: quality of life.

### Risk of Bias and Methodological Quality

#### CHEERS Checklist

Three of the included studies [35,37,40] explicitly stated that the health EE was conducted using the CHEERS guidelines and the 2013 checklist. Ghani et al [35] published these details in the appendix. Scores from our assessment, using the CHEERS statement published in 2022 [31], ranged from 18 [40] to 21 points [35,36]. Overall, the lowest scores were achieved for the criteria related to the title, a health economic analysis plan, distributional effects, and an approach to as well as effects of engagement with patients and others. In particular, those items newly included in the CHEERS 2022 checklist were rarely or not at all addressed in the included studies. Furthermore, criteria relevant to the model-based studies were often poorly documented or not implemented. Visual presentation of the CHEERS assessment can be found in Multimedia Appendix 6.

#### CHEC Checklist Extended

The methodological quality of primarily full trial-based EEs, as judged using the CHEC-checklist, ranged from 14 to 16 fulfilled items (out of 18-20 applicable items), indicating acceptable to good quality. Only in 2 studies were competing alternatives to the evaluated DiHA clearly described. In 2 papers, the ethical and distributional issues were described, although of these, proper elaboration on specific ethical questions regarding DiHA was rarely addressed. For 4 studies, an appropriate time horizon to capture costs and consequences of the interventional arm was found to have been chosen. One study was found to discount future costs and outcomes appropriately. For all other studies, discounting of costs and outcomes was not applicable, as the time horizon was 1 year or shorter. The methodology of measurement in terms of outcomes, costs, and valuing of economic variables was uniformly judged positively. A visual presentation of the CHEC Checklist can be found in Multimedia Appendix 6.

#### Risk of Bias Assessment

The 7 health economic studies, all based on an RCT, were assessed using the RoB 2 tool [29]. The model-based study [40], which relied partly on RCT data, was also included in this assessment. Out of 7 RCTs, 4 were judged to be at high RoB, and 3 clinical trials raised some concerns. Visual presentations of overall RoB can be found in Multimedia Appendix 6.

The randomization process was associated with a low RoB in all clinical trials, as these clinical trials demonstrated proper random allocation sequence generation and concealed allocation. Regarding deviations from the intended intervention, 3 of the 7 studies were judged to have a low RoB, as no deviation from the intended intervention was observed. Almost all authors conducted an intention-to-treat analysis. However, a little more than half of the studies raised some concerns due to deviations from the intended intervention that arose because of the trial context. For around two-thirds of the clinical trials, there was no substantial amount of missing outcome data. However, in 2 trials, there is reason to believe that missing outcome data were related to their true values, leading to a potential for bias in this category. Sensitivity analyses were performed for 5 clinical trials. About one-third of the clinical trials raised high RoB due to the possibility that the assessment of the outcomes was influenced by knowledge of intervention received. Almost one-third of the clinical trials had a low RoB in the selection of the reported results, as a pre-specified protocol was provided and data produced were analyzed in accordance with the pre-specified analysis plan. However, more than half of the clinical trials raised some concerns, as it remains unclear if selective reporting occurred due to missing or not accessible analysis plans.

## Discussion

### Principal Results

Based on the defined inclusion and exclusion criteria, a total of only 7 full health economic studies of DiHA were identified. More than half of the included studies concluded that the app evaluated was cost-effective. Because of the broad scope of this analysis, there is a risk of oversimplification, as the included studies cover a wide range of conditions that differ significantly in their underlying mechanisms, patient populations, and intervention objectives. Grouping these diverse indications under the umbrella of DiHA does not imply a common mechanism of effect. Rather, this review provides a basis for drawing conclusions about the field as a whole by offering a first cross-indication synthesis of cost-effectiveness evidence for independently usable DiHA. However, potential publication bias should be considered, as negative results of health economic studies may not have been published [41]. Notably, only one of the included studies [40] was funded by industry. Only 2 studies used a modeling approach for EE, with one of the studies primarily based on RCT data. All other studies were trial-based EEs. The quality of the studies assessed using the CHEERS 2022 checklist and the CHEC checklist was classified as good on average. However, most RCTs whose data were used for EE were at high RoB. Overall, a good trial design is required to decide whether a given trial is a suitable vehicle for an EE; for example, it is unclear if the study design is capable of providing unbiased answers to the clinical question [42]. Applying the consensus on health economic criteria (CHEC) instrument revealed several difficulties and patterns, some of which may be attributed to CHEC not being optimized for DiHA research.

### Comparison With Prior Work

No systematic review specifically focusing on health EEs of DiHA that can be used independently by patients and without additional sensors or devices could be found. However, there are reviews that have assessed either mHealth interventions, apps in connection with other therapy components, or other adaptations. For example, Iribarren et al [24] examined the economic evidence of mHealth interventions and found that 74.3% of the included interventions were cost-effective, which is a slightly higher percentage compared to our results. Jiang et al [43] examined the cost-effectiveness of digital health interventions for cardiovascular disease and found that about half of the interventions examined achieved dominant or acceptable ICERs in terms of cost per QALY, which is lower than the proportion identified in this paper. However, whether the costs per QALY represent cost-effectiveness depends on the willingness to pay for extra QALYs, which varies in different countries. A systematic review by La Torre-Díez et al [25] examined cost-effectiveness and CUAs of telemedicine, electronic, and mobile health care systems. Their results showed a heterogeneous picture of the economic evidence. However, the authors were able to show that most of the included studies were cost-effective.

In general, there is growing interest in the topic of the cost-effectiveness of DiHA. Most investors in digital health companies recognize that demonstrating value for money is crucial for the success of digital health apps. Positive evidence on health outcomes alone may be insufficient. It’s essential to demonstrate clear, tangible benefits to the health care system to secure both reimbursement for digital health apps and support for scalable adoption [44]. Most of the included studies were published in recent years, targeting a wide range of indications and medical areas. All studies included CUAs, 3 [35-37] additionally included an evaluation based on clinical parameters and therefore CEAs. This finding is only partially consistent with the literature. Iribarren et al [24] mainly found CEAs (64%, n=25). However, according to Boehler [45], the choice of CUA offers the greatest degree of freedom for economic studies of eHealth interventions, indicating its prevalence in current app evaluation practices. All studies were based on RCTs, but one was primarily model-based [40]. Accordingly, the internal validity can be classified as high, and the external validity as low due to the lack of model-based studies and the moderate quality of hybrid studies. In the literature, a naturalistic design is usually recommended as the gold standard for eHealth interventions to enhance the generalizability to patients not involved in the study [46].

The sample sizes of the study-based evaluations were relatively small. De La Torre-Díez et al [25] concluded in their systematic review that the included studies had low participant numbers, which was seen as a major limitation of the EEs. Hazel et al [47] also address the importance of sample size, particularly regarding economies of scale. Small sample sizes may lead to lower estimated cost savings compared to larger samples and, potentially, to an underestimation of costs related to utilization, as higher utilization can increase higher provision costs. Accordingly, this issue should be considered in future evaluations of DiHA.

The time horizons considered in the study-based evaluations are primarily linked to the durations of the respective RCTs and range from 4 months to 1 year. Typically, trial follow-up periods are shorter than the period needed to fully observe the differences in health effects and use of health care resources between interventions being compared. Therefore, time horizon bias presents a significant challenge in EEs based on clinical trials [42]. In general, a longer time horizon is recommended for high-quality studies, or at least the period should be appropriate to the disease in question [48,49]. However, the rapidly evolving nature of apps and the often short periods of use might render long periods unrealistic. One possible approach to address this could be to follow up on studies or to supplement clinical study data with real-world data [50]. In the reviews by Rinaldi et al [51] and Iribarren et al [24] considerably longer observation periods were reported. However, both reviews did not exclusively focus on apps, which suggests that the choice of time horizons is related to the type of intervention.

The comparison groups of the included studies predominantly consisted of standard care or care-as-usual. The frequent use of standard care as the comparator highlights the challenges in determining an appropriate control group and indicates that health care apps tend to be seen as an addition, a delay, or a first step before other therapies. In some cases, these apps may also be used to help patients in tapering off certain therapies, serve as an escalation of treatment, or even act as an alternative to more invasive interventions, such as additional medication, surgery (eg, bariatric surgery), or rehabilitation programs [52,53]. For apps in particular, it is recommended to choose a perspective that also considers non-medical effects, thus adopting the societal perspective [16], as it was done in 3 of the included studies.

The assessment of the RoB revealed that the RCTs underlying the health economic studies should be viewed with some or high concern regarding bias. The results of RoB 2 show that there is a need for improvement in outcome measurement regarding the RoB due to open label or non-blinding. RCTs in DiHA could make better use of the digital space for blinding, but the implementation of blinding in DiHA is difficult [24,54-56]. DiHA studies also have the potential to minimize bias risks due to their digital nature, in which multiple study sites or even multinational trials are easier to implement. However, these options were not used in the included studies. Despite this, the assessment of study quality showed that the overall quality can be categorized as good.

### Limitations

A few limitations of this study should be noted. First, due to the inclusion and exclusion criteria focusing solely on complete health economic studies, it was not possible to investigate whether novel modeling frameworks regarding health economic studies [57] are already being applied to apps or whether further approaches exist. However, since complete health economic studies are considered to be of high methodological quality and provide a foundational overview of current modeling practices, this limitation can be addressed in future research. In addition, the inclusion criteria were strict, as only studies with indication-specific apps were included. This may have resulted in the exclusion of certain apps, highlighting the need for future studies to include broader apps.

Second, literature searches have inherent limitations. Due to the limited selection of databases, not all publications may have been identified [26]. This is particularly true for apps, which are novel interventions and may lack standardized keywords. This challenge is further compounded by the absence of an internationally standardized definition for DiHA.

Third, statements regarding the current practice of modeling should be treated with caution due to the small number of studies included. However, by conducting a systematic literature search in relevant specialized databases, it can be assumed that many published studies could be identified. Given the increasing interest in it, it is expected that many more studies will emerge in the coming years, allowing for updates to this research.

### Conclusions

Given the current lack of high-quality, condition-specific EEs, the broad, exploratory synthesis of this study is both a necessary and valuable first step, as it serves to highlight the emerging potential as well as the methodological gaps in the field of independently usable apps. It could be shown that evaluated apps cover a wide range of conditions. Although 4 out of 7 publications concluded that the app was likely to be cost-effective, this review does not provide a basis for drawing conclusions about the field of DiHA as a whole. Rather, it shows a first cross-indication synthesis of cost-effectiveness evidence for independently usable DiHA, which is an increasingly important category of interventions, particularly in the context of reimbursement and health policy. Nevertheless, health EE is not yet a standard component of evidence generation for digital health apps. Given the increasing demands on health care systems, rising costs, and labor shortages, digital health apps present promising solutions. Still, manufacturers must demonstrate value for money to ensure digital health apps’ integration in health systems. To help innovators and manufacturers conduct health EEs and provide decision-makers with the necessary evidence, more robust information on evidence requirements, including, for instance, accepted study designs or modeling practices, is crucial.

## Supplementary material

10.2196/68349 Multimedia Appendix 1 PRISMA checklist.

10.2196/68349 Multimedia Appendix 2 Search strategies.

10.2196/68349 Multimedia Appendix 3 List of excluded studies

10.2196/68349 Multimedia Appendix 4 Funding and competing interest of included economic evaluations.

10.2196/68349 Multimedia Appendix 5 Overview of all outcomes and costs.

10.2196/68349 Multimedia Appendix 6 Risk of bias and methodological quality of included economic evaluations.

## Abbreviations

CEA: cost-effectiveness analysis
CHEC: Consensus on Health Economic Criteria
CHEERS: Consolidated Health Economic Evaluation Reporting Standards
CUA: cost-utility analysis
EE: economic evaluation
HTA: health technology assessment
ICER: incremental cost-effectiveness ratio
PRISMA: Preferred Reporting Items for Systematic Reviews and Meta-Analyses
QALY: quality-adjusted life year
RCT: randomized controlled trial
RoB: risk of bias
RoB 2: Cochrane risk of bias tool 2
