# Supplementary material for: An Overview on Methods, Evidence, and Study Quality of Health Economic Evaluation Studies for Independently Usable Digital Health Apps: Rapid Review
Source: J Med Internet Res. 2025 Aug 19;27:e68349. doi: 10.2196/68349 (PMC12364420; doi:10.2196/68349)
Supplement: Multimedia Appendix 2 [file jmir-v27-e68349-s002.docx]

### Appendix 2 – Search strategies

1. **PubMed via Pubmed**

#1 “Mobile phone“[tiab]

#2 Smartphone[MeSH Terms]

#3 Smartphone[tiab]

#4 Cell Phone[Mesh:noexp]

#5 “Cell phone”[tiab]

#6 “mobile-based system”[tiab]

#7 “digital health application*”[tiab]

#8 Mobile Applications[Mesh Terms]

#9 “Mobile application*”[tiab]

#10 “Application software”[tiab]

#11 “Mobile App”[tiab]

#12 “Health App”[tiab]

#13 “Medical App”[tiab]

#14 App[tiab]

#15 Apps[tiab]

#16 “mobile Health”[tiab]

#17 mHealth[tiab]

#18 #1 OR #2 OR #3 OR #4 OR #5 OR #6 OR #7 OR #8 OR #9 OR #10 OR #11 OR #12 OR #13 OR #14 OR #15 OR #16 OR #17

#19 “health economic stud*”[tiab]

#20 Cost-Benefit Analysis[Mesh Terms]

#21 “Cost-Benefit Analys*”[tiab]

#22 “Cost-Utility Analys*”[tiab]

#23 “Cost-Effectiveness Analys*”[tiab]

#24 “Cost-Minimization Analys*“[tiab]

#25 “Cost-Minimisation Analys*“[tiab]

#26 “cost-effectiveness”[tiab]

#27 “cost-effective”[tiab]

#28 #19 OR #20 OR #21 OR #22 OR #23 OR #24 OR #25 OR #26 OR #27

#29 #18 AND #28

Filter: 2008-April 2023

1. **Web of Science**

#1 TS=(“Mobile Phone” OR Smartphone OR “Cell Phone” OR “mobile-based system”)

#2 TS=(“digital health application*” OR “Mobile Application*” OR “Application software” OR “Mobile App” OR “Health App” OR “Medical App” OR App OR Apps)

#3 TS=(“mobile Health” OR mHealth)

#4 #1 OR #2 OR #3

#5 TS=(“health economic stud*” OR “Cost-Benefit Analys?s” OR “Cost-Utility Analys?s” OR “Cost-Effectiveness Analys?s” OR “Cost-Minimi?ation Analys?s” OR “cost-effectiveness” OR “cost-effective”)

#6 #4 AND #5

Filter: 2008-April 2023

1. **EconBiz**

#1 “Mobile phone“

#2 Mobiltelefon

#3 Smartphone

#4 “tragbares Telefon”

#5 Handy

#6 “Handy-Telefon”

#7 “Cell phone”

#8 “mobile-based system”

#9 “digital health application”

#10 “digitale Gesundheitsanwendung”

#11 “Mobile application”

#12 “Application software”

#13 “Mobile App”

#14 “Health App”

#15 “Gesundheits-App”

#16 “Medical App”

#17 “Medizin-App”

#18 App

#19 Apps

#20 “mobile Health”

#21 mHealth

#22 #1 OR #2 OR #3 OR #4 OR #5 OR #6 OR #7 OR #8 OR #9 OR #10 OR #11 OR #12 OR #13 OR #14 OR #15 OR #16 OR #17 OR #18 OR #19 OR #20 OR #21

#23 “health economic study”

#24 “gesundheitsökonomische Studie“

#25 “Cost-Benefit Analysis”

#26 “Cost-Benefit Analyses”

#27 “Kosten-Nutzen-Analyse”

#28 “Cost-Utility Analysis”

#29 “Cost-Utility Analyses”

#30 “Kosten-Nutzwert-Analyse”

#31 Nutzwertanalyse

#32 “Kosten-Nutzwertanalyse”

#33 “Cost-Effectiveness Analysis”

#34 “Cost-Effectiveness Analyses”

#35 “Kosten-Effektivitäts-Analyse”

#36 Kosteneffektivitätsanalyse

#37 “Kosten-Wirksamkeits-Analyse”

#38 “Cost-Minimization Analysis“

#39 “Cost-Minimization Analyses”

#40 “Cost-Minimisation Analysis“

#41 “Cost-Minimisation Analyses”

#42 “Kosten-Minimierungs-Analyse”

#43 Kostenminimierungsanalyse

#44 “cost-effectiveness”

#45 Kosteneffektivität

#46 “cost-effective”

#47 kosteneffektiv

#48 #23 OR #24 OR #25 OR #26 OR #27 OR #28 OR #29 OR #30 OR #31 OR #32 OR #33 OR #34 OR #35 OR #36 OR #37 OR #38 OR #39 OR #40 OR #41 OR #42 OR #43 OR #44 OR #45 OR #46 OR #47

#49 #22 AND #48

Filter: 2008-April 2023

1. **Cochrane Library**

#1 (“Mobile phone”):ti,ab,kw

#2 MeSH descriptor: [Smartphone] explode all trees

#3 (Smartphone):ti,ab,kw

#4 MeSH descriptor: [Cell Phone] this term only

#5 (“Cell phone”):ti,ab,kw

#6 (“mobile-based system”):ti,ab,kw

#7 (“digital health application”):ti,ab,kw

#8 MeSH descriptor: [Mobile applications] explode all trees

#9 (“Mobile Application”):ti,ab.kw

#10 (“Application software”):ti,ab,kw

#11 (“Mobile App”):ti,ab,kw

#12 (“Health App”):ti,ab,kw

#13 (“Medical App”):ti,ab,kw

#14 (App):ti,ab,kw

#15 (Apps):ti,ab,kw

#16 (“mobile Health”):ti,ab,kw

#17 (mHealth):ti,ab,kw

#18 #1 OR #2 OR #3 OR #4 OR #5 OR #6 OR #7 OR #8 OR #9 OR #10 OR #11 OR #12 OR #13 #14 OR #15 OR #16 OR #17

#19 (“health economic study”):ti,ab,kw

#20 MeSH descriptor: [Cost-Benefit Analysis] explode all trees

#21 (“Cost-Benefit Analysis”):ti,ab,kw

#22 (“Cost-Utility Analysis”):ti,ab,kw

#23 (“Cost-Effectiveness Analysis”):ti,ab,kw

#24 (“Cost-Minimization Analysis”):ti,ab,kw

#25 (“cost-effectiveness”):ti,ab,kw

#26 (“cost-effective”):ti,ab,kw

#27 #19 OR #20 OR #21 OR #22 OR #23 OR #24 OR #25 OR #26

#28 #18 AND #27

Filter: 2008-April 2023
