# Supplementary material for: An Overview on Methods, Evidence, and Study Quality of Health Economic Evaluation Studies for Independently Usable Digital Health Apps: Rapid Review
Source: J Med Internet Res. 2025 Aug 19;27:e68349. doi: 10.2196/68349 (PMC12364420; doi:10.2196/68349)
Supplement: Multimedia Appendix 3 [file jmir-v27-e68349-s003.docx]

### Appendix 3 – List of excluded studies

Where more than one exclusion reason applied, we chose the first reason according to the PICO scheme. Studies that seem to meet the inclusion criteria, but which were excluded, are marked as “close misses” and an additional explanation for their exclusion is provided.

| No. | Reference | Reason for exclusion |
| --- | --- | --- |
| 1. | Adamson BJ, Jain R, Garrison LP, Lutz BR. SMARTPHONE-ENHANCED RAPID INFLUENZA TESTS: A COST-EFFECTIVENESS ANALYSIS. *VALUE IN HEALTH*. 2016;19(3):A302. | Intervention |
| 2. | Agnihothri S, Cui L, Delasay M, Rajan B. The value of mHealth for managing chronic conditions. *HEALTH CARE MANAGEMENT SCIENCE*. 2020;23(2):185-202. | Study type |
| 3. | Ambrens M, van Schooten KS, Lung T, et al. Economic evaluation of the e-Health StandingTall balance exercise programme for fall prevention in people aged 70 years and over. *Age and ageing*. 2022;51(6). doi:10.1093/ageing/afac130 | Intervention |
| 4. | Augustovski F, Palacios A, Beratarrechea A, et al. Cost-effectiveness analysis of a randomized trial of an mhealth intervention to improve cardiometabolic profile in prehypertensive subjects from low-resource urban settings in Latin America. *Value in health*. 2017;20(9):A916‐. | No fulltext |
| 5. | Birkemeyer R, Müller A, Wahler S, Von Der Schulenburg JM. A cost-effectiveness analysis model of Preventicus atrial fibrillation screening from the point of view of statutory health insurance in Germany. *Health Econ Rev*. 2020;10(1):16. doi:10.1186/s13561-020-00274-z | Intervention |
| 6. | Boels A, Vos R, Dijkhorst-Oei LT, Rutten G. Smartphone triggered diabetes self-management education and support in insulin treated type 2 diabetes patients: results of the randomised TRIGGER study. *Diabetologia*. 2018;61:S403‐S404. | Intervention |
| 7. | Bone JN, Khowaja AR, Vidler M, et al. Economic and cost-effectiveness analysis of the Community-Level Interventions for Pre-eclampsia (CLIP) trials in India, Pakistan and Mozambique. *BMJ GLOBAL HEALTH*. 2021;6(5). | Intervention |
| 8. | Boodoo C, Zhang Q, Ross HJ, Alba AC, Laporte A, Seto E. Evaluation of a Heart Failure Telemonitoring Program Through a Microsimulation Model: Cost-Utility Analysis. *JOURNAL OF MEDICAL INTERNET RESEARCH*. 2020;22(10). | Intervention |
| 9. | Bowser DM, Shepard DS, N, et al. Cost Effectiveness of Mobile Health for Antenatal Care and Facility Births in Nigeria. *ANNALS OF GLOBAL HEALTH*. 2018;84(4):592-602. | Patient |
| 10. | Brezing CA, Brixner DI. The Rise of Prescription Digital Therapeutics in Behavioral Health. *Advances in therapy*. 2022;39(12):5301-5306. doi:10.1007/s12325-022-02320-0 | Study type |
| 11. | Browne JD, Vaninetti M, Giard D, Kostas K, Dave A. An Evaluation of a Mobile App for Chronic Low Back Pain Management: Prospective Pilot Study. *JMIR formative research*. 2022;6(10):e40869. doi:10.2196/40869 | Study type |
| 12. | Burn E, Nghiem S, Jan S, et al. Cost-effectiveness of a text message programme for the prevention of recurrent cardiovascular events. *Heart*. 2017;103(12):893.1-894. doi:10.1136/heartjnl-2016-310195 | Intervention |
| 13. | Cano Martín JA, Martínez-Pérez B, De La Torre-Díez I, López-Coronado M. Economic Impact Assessment from the Use of a Mobile App for the Self-management of Heart Diseases by Patients with Heart Failure in a Spanish Region. *J Med Syst*. 2014;38(9):96. doi:10.1007/s10916-014-0096-z | Patient |
| 14. | Catuara-Solarz S, Skorulski B, Estella-Aguerri I, et al. The Efficacy of “Foundations,” a Digital Mental Health App to Improve Mental Well-being During COVID-19: Proof-of-Principle Randomized Controlled Trial. *JMIR mHealth and uHealth*. 2022;10(7):e30976. doi:10.2196/30976 | Study type |
| 15. | Cunningham SG, Stoddart A, Wild SH, Conway NJ, Gray AM, Wake DJ. Cost-Utility of an Online Education Platform and Diabetes Personal Health Record: Analysis Over Ten Years. *J Diabetes Sci Technol*. 2023;17(3):715-726. doi:10.1177/19322968211069172 | Intervention |
| 16. | Dahlberg K, Philipsson A, Hagberg L, Jaensson M, Hälleberg-Nyman M, Nilsson U. Cost-effectiveness of a systematic e-assessed follow-up of postoperative recovery after day surgery: a multicentre randomized trial. *British Journal of Anaesthesia*. 2017;119(5):1039-1046. doi:10.1093/bja/aex332 | Intervention |
| 17. | Daly AT, Deshmukh AA, Vidrine DJ, et al. Cost-effectiveness analysis of smoking cessation interventions using cell phones in a low-income population. *Tob Control*. Published online June 9, 2018:tobaccocontrol-2017-054229. doi:10.1136/tobaccocontrol-2017-054229 | Intervention |
| 18. | Dana R, Sullivan S, MacGowan RJ, et al. Engaging Black or African American and Hispanic or Latino Men Who Have Sex With Men for HIV Testing and Prevention Services Through Technology: Protocol for the iSTAMP Comparative Effectiveness Trial. *JMIR research protocols*. 2023;12:e43414. doi:[10.2196/43414](https://doi.org/10.2196/43414) | Study type |
| 19. | Dawson J, Howell M, Howard K, et al. Cost‐effectiveness of a mobile phone text messaging program (KIDNEYTEXT) targeting dietary behaviours in people receiving haemodialysis. *J Human Nutrition Diet*. 2022;35(5):765-773. doi:10.1111/jhn.12937 | Intervention |
| 20. | Ebert DD, KÃ¤hlke F, Buntrock C, et al. A health economic outcome evaluation of an internet-based mobile-supported stress management intervention for employees. *Scand J Work Environ Health*. Published online November 16, 2017. doi:10.5271/sjweh.3691 | Intervention |
| 21. | Ellmann S, Maryschok M, Schöffski O, Emmert M. The German COVID-19 Digital Contact Tracing App: A Socioeconomic Evaluation. *IJERPH*. 2022;19(21):14318. doi:10.3390/ijerph192114318 | Intervention |
| 22. | Fatoye F, Maikudi-Olofu L, Gebrye T, Fatoye C, Mbada C. POSC72 Clinical and Cost Effectiveness of a Clinic-Based and Two Digital Applications of McKenzie Therapy for Chronic Low-Back Pain. *Value in Health*. 2022;25(1):S100. doi:10.1016/j.jval.2021.11.477 | Patient |
| 23. | Fawsitt CG, Meaney S, Greene RA, Corcoran P. Surgical Site Infection after Caesarean Section? There Is an App for That: Results from a Feasibility Study On Costs and Benefits. *Ir Med J*. 2017;110(9):635. | Study type |
| 24. | Fawsitt C, Meaney S, Corcoran P. A cost-benefit analysis of a mobile application to monitor surgical wound post-caesarean section: a pilot-based, feasibility study. *BJOG-AN INTERNATIONAL JOURNAL OF OBSTETRICS AND GYNAECOLOGY*. 2016;123(1):55. | Study type |
| 25. | Fottrell E, Ahmed N, Morrison J, et al. Community groups or mobile phone messaging to prevent and control type 2 diabetes and intermediate hyperglycaemia in Bangladesh (DMagic): a cluster-randomised controlled trial. *The lancet Diabetes & endocrinology*. 2019;7(3):200‐212. | Intervention |
| 26. | Fritzen K, Basinska K, Rubio-Almanza M, et al. Pan-European Economic Analysis to Identify Cost Savings for the Health Care Systems as a Result of Integrating Glucose Monitoring Based Telemedical Approaches Into Diabetes Management. *Journal of Diabetes Science and Technology*. 2019;13(6):1112‐1122. | Intervention |
| 27. | Gilmer T, Burgos J, Anzaldo-Campos M, Vargas-Ojeda A. Cost-Effectiveness of a Technology-Enhanced Diabetes Care Management Program in Mexico. *Value in health regional issues*. 2019;20:41‐46. | Patient |
| 28. | Gleason LT, Xie R, Wood L, et al. Cost-benefit analysis of a patient engagement technology (PET) in cardiac, thoracic, and colorectal surgery. *AMERICAN JOURNAL OF SURGERY*. 2022;224(3):979-986. doi:10.1016/j.amjsurg.2022.04.030 | No fulltext |
| 29. | Goryakin Y, Aldea A, Lerouge A. Promoting sport and physical activity in Italy: a costeffectiveness analysis of seven innovative public health policies. *annali di igiene medicina preventiva e di comunità*. 2019;(6):614-625. doi:10.7416/ai.2019.2321 | Study type |
| 30. | Grout L, Telfer K, Wilson N, Cleghorn C, Mizdrak A. Prescribing Smartphone Apps for Physical Activity Promotion in Primary Care: Modeling Study of Health Gain and Cost Savings. *J Med Internet Res*. 2021;23(12):e31702. doi:10.2196/31702 | Study type |
| 31. | Guerriero C, Cairns J, Roberts I, Rodgers A, Whittaker R, Free C. The cost-effectiveness of smoking cessation support delivered by mobile phone text messaging: txt2stop. *European journal of health economics*. 2013;14(5):789‐797. | Intervention |
| 32. | Gumbie M, Parkinson B, Dillon H, Bowman R, Song R, Cutler H. Cost-Effectiveness of Screening Preschool Children for Hearing Loss in Australia. *Ear & Hearing*. 2022;43(3):1067-1078. doi:10.1097/AUD.0000000000001134 | Patient |
| 33. | Gumley AI, Bradstreet S, Ainsworth J, et al. Digital smartphone intervention to recognise and manage early warning signs in schizophrenia to prevent relapse: the EMPOWER feasibility cluster RCT. Health technology assessment (Winchester, England). 2022;26(27):1-174. doi:10.3310/hlze0479 | Intervention |
| 34. | Haycocks S, Cameron R, Edge M, Budd J, Chadwick P. Implementation of a novel mHealth application for the management of people with diabetes and recently healed foot ulceration: A feasibility study. Digital health. 2022;8:20552076221142103. doi:10.1177/20552076221142103 | Intervention |
| 35. | Hill J, McGinn J, Cairns J, Free C, Smith C. A Mobile Phone-Based Support Intervention to Increase Use of Postabortion Family Planning in Cambodia: Cost-Effectiveness Evaluation. JMIR mHealth and uHealth. 2020;8(2):e16276‐. | Intervention |
| 36. | Hunchangsith P, Barendregt JJ, Vos T, Bertram M. Cost-Effectiveness of Various Tuberculosis Control Strategies in Thailand. *Value in Health*. 2012;15(1):S50-S55. doi:10.1016/j.jval.2011.11.006 | Intervention |
| 37. | Islam SMS. A9640 Effectiveness and cost effectiveness of a mobile phone text messaging intervention for prevention of cardiovascular risk factors among patients with type 2 diabetes: a randomized controlled trial. *Journal of Hypertension*. 2018;36:e315. doi:10.1097/01.hjh.0000549285.14579.2e | Intervention |
| 38. | Islam SMS, Peiffer R, Chow CK, et al. Cost-effectiveness of a mobile-phone text messaging intervention on type 2 diabetes-A randomized-controlled trial. *HEALTH POLICY AND TECHNOLOGY*. 2020;9(1):79-85. | Intervention |
| 39. | Kapoor P, Chowdhry A, Sengar P, Mehta A. Development, testing, and feasibility of a customized mobile application for obstructive sleep apnea (OSA) risk assessment: A hospital-based pilot study. *Journal of oral biology and craniofacial research*. 2022;12(1):109-115. doi:10.1016/j.jobcr.2021.11.004 | Study type |
| 40. | Kaul R, Akhoon N. Use of the Bone Ninja Mobile Application as a Pre-operative Assessment and Simulation Tool in Patients Undergoing High Tibial Osteotomy. *Revista brasileira de ortopedia*. 2022;57(1):89-95. doi:10.1055/s-0040-1716761 | Study type |
| 41. | Kazi DS, Prabhakaran D, Bolger AF. Rising above the rhetoric: mobile applications and the delivery of cost-effective cardiovascular care in resource-limited settings. *Future Cardiology*. 2015;11(1):1-4. doi:10.2217/fca.14.74 | Study type |
| 42. | Kock JH de, Latham HA, Cowden RG, et al. Brief Digital Interventions to Support the Psychological Well-being of NHS Staff During the COVID-19 Pandemic: 3-Arm Pilot Randomized Controlled Trial. *JMIR mental health*. 2022;9(4):e34002. doi:10.2196/34002 | Study type |
| 43. | Kodama T, Tamura Y, Komori T, Kataoka M, Igura K, Hashimoto T. A Pilot Randomized Controlled Trial of a Text Message Intervention to Promote Help Seeking for Psychiatric Outpatients. *Computers, informatics, nursing*. 2020;39(3):154‐161. | No fulltext |
| 44. | Lewkowicz D, Wohlbrandt AM, Bottinger E. Digital Therapeutic Care Apps With Decision-Support Interventions for People With Low Back Pain in Germany: Cost-Effectiveness Analysis. *JMIR Mhealth Uhealth*. 2022;10(2):e35042. doi:10.2196/35042 | Intervention |
| 45. | Li J, Sun L, Hou Y, Chen L. Cost-Effectiveness Analysis of a Mobile-Based Intervention for Patients with Type 2 Diabetes Mellitus. *INTERNATIONAL JOURNAL OF ENDOCRINOLOGY*. 2021;2021. | Intervention |
| 46. | Lim ML, Tran M, van Schooten KS, et al. A Self-Guided Online Cognitive Behavioural Therapy to Reduce Fear of Falling in Older People: a Randomised Controlled Trial. *International journal of behavioral medicine*. Published online January 2022. doi:10.1007/s12529-022-10105-6 | Study type |
| 47. | Linardon J, Shatte A, Rosato J, Fuller-Tyszkiewicz M. Efficacy of a transdiagnostic cognitive-behavioral intervention for eating disorder psychopathology delivered through a smartphone app: a randomized controlled trial. *Psychological medicine*. 2022;52(9):1679-1690. doi:10.1017/s0033291720003426 | Study type |
| 48. | Loohuis A, Worp van der, Wessels N, et al. APP-BASED TREATMENT FOR FEMALE URINARY INCONTINENCE AS EFFECTIVE AND COST-EFFECTIVE IN COMPARISON TO CARE AS USUAL IN PRIMARY CARE: A PRAGMATIC RANDOMIZED CONTROLLED TRIAL. *NEUROUROLOGY AND URODYNAMICS*. 2020;39(2):S366-S367. | Study type |
| 49. | Luo X, Xu W, Ming WK, et al. Cost-Effectiveness of Mobile Health–Based Integrated Care for Atrial Fibrillation: Model Development and Data Analysis. *J Med Internet Res*. 2022;24(4):e29408. doi:10.2196/29408 | Intervention |
| 50. | Luxton DD, Hansen RN, Stanfill K. Mobile app self-care versus in-office care for stress reduction: a cost minimization analysis. *J Telemed Telecare*. 2014;20(8):431-435. doi:10.1177/1357633X14555616 | Study type |
| 51. | Mandal PK, Saharan S, Khan SA, James M. Apps for Dementia Screening: A Cost-effective and Portable Solution. *JAD*. 2015;47(4):869-872. doi:10.3233/JAD-150255 | Study type |
| 52. | McBeth PB, Hamilton T, Kirkpatrick AW. Cost-Effective Remote iPhone-Teathered Telementored Trauma Telesonography. *Journal of Trauma: Injury, Infection & Critical Care*. 2010;69(6):1597-1599. doi:10.1097/TA.0b013e3181e61ea9 | Study type |
| 53. | Meenakshi R, Ponnusamy R, Alghamdi S, Khalaf OI, Alotaibi Y. Development of Mobile App to Support the Mobility of Visually Impaired People. *CMC-COMPUTERS MATERIALS & CONTINUA*. 2022;73(2):3473-3495. doi:10.32604/cmc.2022.028540 | Intervention |
| 54. | Miranda RN, Bhuiya AR, Thraya Z, et al. An Electronic Patient-Reported Outcomes Tool for Older Adults With Complex Chronic Conditions: Cost-Utility Analysis. *JMIR Aging*. 2022;5(2):e35075. doi:10.2196/35075 | Intervention |
| 55. | Morphew T, Scott L, Li M, et al. Mobile Health Care Operations and Return on Investment in Predominantly Underserved Children with Asthma: The Breathmobile Program. *Population Health Management*. 2013;16(4):261-269. doi:10.1089/pop.2012.0060 | Intervention |
| 56. | Mujcic A, Blankers M, Boon B, et al. Effectiveness, Cost-effectiveness, and Cost-Utility of a Digital Alcohol Moderation Intervention for Cancer Survivors: Health Economic Evaluation and Outcomes of a Pragmatic Randomized Controlled Trial. *J Med Internet Res*. 2022;24(2):e30095. doi:10.2196/30095 | Intervention |
| 57. | O’Sullivan EJ, Kennelly MA, Rokicki S, Ainscough K, McAuliffe FM. Cost-effectiveness of a mobile health-supported lifestyle intervention for preventing gestational diabetes mellitus. *AMERICAN JOURNAL OF OBSTETRICS AND GYNECOLOGY*. 2018;218(1):S380-S381. | Intervention |
| 58. | O’Sullivan EJ, Rokicki S, Kennelly M, Ainscough K, McAuliffe FM. Cost-effectiveness of a mobile health-supported lifestyle intervention for pregnant women with an elevated body mass index. *Int J Obes*. 2020;44(5):999-1010. doi:10.1038/s41366-020-0531-9 | Intervention |
| 59. | Oostingh EC, Ophuis RH, Koster MP, et al. Mobile Health Coaching on Nutrition and Lifestyle Behaviors for Subfertile Couples Using the Smarter Pregnancy Program: Model-Based Cost-Effectiveness Analysis. *JMIR Mhealth Uhealth*. 2019;7(10):e13935. doi:10.2196/13935 | Intervention |
| 60. | Ostermann J, Njau B, Masaki M, et al. Feasibility, Acceptability, and Potential Cost-Effectiveness of a Novel Mobile Phone Intervention to Promote Human Immunodeficiency Virus Testing Within Social Networks in Tanzania. *Sexual Trans Dis*. 2022;49(11):778-781. doi:10.1097/OLQ.0000000000001611 | Intervention |
| 61. | Paganini S, Lin J, Kählke F, et al. A guided and unguided internet- and mobile-based intervention for chronic pain: health economic evaluation alongside a randomised controlled trial. *BMJ open*. 2019;9(4):e023390. | Intervention |
| 62. | Patnaik L, Panigrahi S, Kumar eep, et al. Effectiveness of Mobile Application for Promotion of Physical Activity Among Newly Diagnosed Patients of Type II Diabetes - A Randomized Controlled Trial. *International journal of preventive medicine*. 2022;13:54. doi:10.4103/ijpvm.IJPVM_92_20 | Study type |
| 63. | Pelle T, Bevers K, Van Den Hoogen F, Van Der Palen J, Van Den Ende C. Economic evaluation of the dr. Bart app in people with knee and/or hip osteoarthritis. *Annals of the rheumatic diseases*. 2020;79:1947‐. | Study type |
| 64. | Pelle T, Bevers K, van den Hoogen F, van der Palen J, van den Ende E. Economic evaluation of the Dr. Bart app in people with knee and/or hip osteoarthritis. *Osteoarthritis and cartilage*. 2021;29:S386‐. | Study type |
| 65. | Pooni A, Brar MS, Anpalagan T, et al. Home to Stay: A Randomized Controlled Trial Evaluating the Effect of a Post-discharge Mobile App to Reduce 30-Day Re-admission Following Elective Colorectal Surgery. *Annals of surgery*. Published online January 2022. doi:10.1097/sla.0000000000005527 | No fulltext |
| 66. | Price H, Waters AM, Mighty D, et al. Texting appointment reminders reduces ‘Did not Attend’ rates, is popular with patients and is cost-effective. *Int J STD AIDS*. 2009;20(2):142-143. doi:10.1258/ijsa.2008.008448 | Intervention |
| 67. | Priebe S, Golden E, Kingdon D, et al. Effective patient–clinician interaction to improve treatment outcomes for patients with psychosis: a mixed-methods design. *Programme Grants Appl Res*. 2017;5(6):1-160. doi:10.3310/pgfar05060 | Intervention |
| 68. | Rahim MJ, Schwebel DC, Hasan R, Griffin R, Sen B. Cost-benefit analysis of a distracted pedestrian intervention. *INJURY PREVENTION*. doi:10.1136/ip-2022-044740 | No fulltext |
| 69. | Reback CJ, Fletcher JB, Leibowitz AA. Cost effectiveness of text messages to reduce methamphetamine use and HIV sexual risk behaviors among men who have sex with men. *Journal of Substance Abuse Treatment*. 2019;100:59-63. doi:10.1016/j.jsat.2019.02.006 | Intervention |
| 70. | Rogers B, Somé JW, Bakun P, et al. Validation of the INDDEX24 mobile app *v* . a pen-and-paper 24-hour dietary recall using the weighed food record as a benchmark in Burkina Faso. *Br J Nutr*. 2022;128(9):1817-1831. doi:10.1017/S0007114521004700 | Study type |
| 71. | Rondina R, Hong M, Sarma S, Mitchell M. Is it worth it? Cost-effectiveness analysis of a commercial physical activity app. *BMC Public Health*. 2021;21(1):1950. doi:10.1186/s12889-021-11988-y | Population |
| 72. | Ryan D, Price D, Musgrave S, et al. Clinical and cost effectiveness of mobile phone supported self monitoring of asthma: multicentre randomised controlled trial. *BMJ (online)*. 2012;344(7854). https://www.cochranelibrary.com/central/doi/10.1002/central/CN-00979471/full | Patient |
| 73. | Skov-Ettrup LS, Dalum P, Bech M, Tolstrup JS. The effectiveness of telephone counselling and internet- and text-message-based support for smoking cessation: results from a randomized controlled trial. *ADDICTION*. 2016;111(7):1257-1266. | Intervention |
| 74. | Steegers-Theunissen R, Hoek A, Groen H, et al. Pre-Conception Interventions for Subfertile Couples Undergoing Assisted Reproductive Technology Treatment: Modeling Analysis. *JMIR Mhealth Uhealth*. 2020;8(11):e19570. doi:10.2196/19570 | Intervention |
| 75. | Van Reijen M, Vriend I, van Mechelen W, Verhagen E. Preventing recurrent ankle sprains: is the use of an App more cost-effective than a printed Booklet? Results of a RCT. *Scandinavian journal of medicine & science in sports*. 2018;28(2):641‐648. | Intervention |
| 76. | Vidmar AP, Pretlow R, Borzutzky C, et al. An addiction model‐based mobile health weight loss intervention in adolescents with obesity. *Pediatric Obesity*. 2019;14(2):e12464. doi:10.1111/ijpo.12464 | Intervention |
| 77. | Wahler S, Birkemeyer R, Alexopoulos D, Siudak Z, Müller A, Von Der Schulenburg JM. Cost-effectiveness of a photopethysmographic procedure for screening for atrial fibrillation in 6 European countries. *Health Econ Rev*. 2022;12(1):17. doi:10.1186/s13561-022-00362-2 | Intervention |
| 78. | Watanabe Y, Kuroki T, Ichikawa D, Ozone M, Uchimura N, Ueno T. Effect of smartphone-based cognitive behavioral therapy app on insomnia: a randomized, double-blind study. *SLEEP*. doi:10.1093/sleep/zsac270 | No fulltext |
| 79. | Webb CA, Swords CM, Lawrence HR, Hilt LM. Which adolescents are well-suited to app-based mindfulness training? A randomized clinical trial and data-driven approach for personalized recommendations. *Journal of consulting and clinical psychology*. 2022;90(9):655-669. doi:10.1037/ccp0000763 | No fulltext |
| 80. | Yang JY, Wu YW, Chuang W, et al. An Integrated Community-Based Blood Pressure Telemonitoring Program-A Population-Based Observational Study. *ACTA CARDIOLOGICA SINICA*. 2022;38(5):612-622. doi:10.6515/ACS.202209_38(5).20220330A | Study type |
| 81. | Zafari Z, Goldman L, Kovrizhkin K, Muennig PA. The cost-effectiveness of common strategies for the prevention of transmission of SARS-CoV-2 in universities. Schwartzman K, ed. *PLoS ONE*. 2021;16(9):e0257806. doi:10.1371/journal.pone.0257806 | Intervention |
| 82. | Zhang X, Liao H, Shi D, Li X, Chen X, He S. Cost-effectiveness analysis of different hypertension management strategies in a community setting. *Intern Emerg Med*. 2020;15(2):241-250. doi:10.1007/s11739-019-02146-9 | Intervention |
