# Supplementary material for: An Overview on Methods, Evidence, and Study Quality of Health Economic Evaluation Studies for Independently Usable Digital Health Apps: Rapid Review
Source: J Med Internet Res. 2025 Aug 19;27:e68349. doi: 10.2196/68349 (PMC12364420; doi:10.2196/68349)
Supplement: Multimedia Appendix 4 [file jmir-v27-e68349-s004.docx]

### Appendix 4 – Funding and competing interest of included economic evaluations

| **Author (Year** | **Category of funding** | **Funder** | **Competing interests** |
| --- | --- | --- | --- |
| Ekersund et al. (2022) | non-profit | County Council Region Jämtland Härjedalen (JLL-933083, JLL-930732), Northern County Councils (931113), Kamprad Family Foundation for Entrepreneurship, Research, and Charity (20170202). | The logos Tät and Tät.nu are registered as trademarks by the Swedish Patent and Registration office for eContinence AB, a Swedish e-health company founded in July 2021 with the aim of maintaining, spreading, commercializing, and further developing the apps created within the research project Tät.nu (eCont inence. se).  Malin Sjöström and Eva Samuelsson are cofounders and shareholders of eContinence AB and have a potential future financial gain in the app Tät II. Eva Samuelsson is the managing director of eContinence AB. |
| Ghani et al. (2022) | Non-profit | not reported | This study is part of SMART4MD, European Commission, Horizon 2020 project. We greatly acknowledge the financial support. Neither the financial sponsor nor Healthbit Ltd played any role in the design, execution, analysis and interpretation of data data, write-up of the study, or the decision to publish. |
| Loohuis et al. (2022) | non-profit | ZonMw, the Dutch Organisation for Health Research and Development (project number: 837001508).  Sub- funder: P.W. Boer Foundation. | None declared |
| Pelle et al. (2021) | non-profit | Within the INTERREG-programme.  European Union; the Ministry of Economic Affairs, Innovation, Digitalisation and Energy of the State of North Rhine-Westphalia; the Ministry of Economic Affairs and Climate Policy of the Netherlands; and the Dutch Provinces of Gelderland and Limburg | None declared |
| Röhr et al. (2021) | non-profit | Part of study HELP@APP.  Innovationsfonds of the German Federal Joint Committee (Gemeinsamer Bundesausschuss grant 01VSF16033) Innovationsfonds of the German Federal Joint Committee (Gemeinsamer Bundesausschuss grant 01VSF16033) | None declared |
| Sjöström et al. (2017) | non-profit | Swedish Council for Working Life and Social Research, the Region Jämtland Härjedalen, and Visare Norr, Northern County Councils, Sweden. | None declared |
| Song & Kanaoka (2018) | non-profit | docomo Healthcare, Inc. | MS reports personal fees for general honoraria, consulting and lectures, and non-financial support for analysis, writing, and editing from docomo Healthcare, Inc. during the course of the study.  Personal fees were obtained for lectures from ROHTO Pharmaceutical Co. Ltd, Takeda Pharmaceutical Co. Ltd, Otsuka Pharmaceutical Co. Ltd, and Jex Co. Ltd outside the submitted work.  HK is an employee of docomo Healthcare, Inc. A peer reviewer on this manuscript has disclosed that they are an employee of SPD Development Company Ltd, a wholly owned subsidiary of SPD Swiss Precision Diagnostics GmbH, the manufacturer of fertility and pregnancy tests. |
