# Supplementary material for: An Overview on Methods, Evidence, and Study Quality of Health Economic Evaluation Studies for Independently Usable Digital Health Apps: Rapid Review
Source: J Med Internet Res. 2025 Aug 19;27:e68349. doi: 10.2196/68349 (PMC12364420; doi:10.2196/68349)
Supplement: Multimedia Appendix 5 [file jmir-v27-e68349-s005.docx]

### Appendix 5 – Overview of all outcomes and costs

|  | **Effects** | **Costs** |  |
| --- | --- | --- | --- |
| **Author (Year)** | **Outcome measurement** | **Costs included (data source)** | **Results on costs (disaggregated and aggregated form (with CIs))** |
|  |  |  |  |
| **Ekersund et al. (2022)** | - QALY^a^, calculated based on ICIQ-LUTSqol^b^, indication-specific | Costs of the RCT^w^ to determine the benefit, direct medical and non-medical costs (implementation of treatment, incontinence aids, laundry), indirect costs (time for training), from Swedish kronor in euros (2017) | Total cost: €741.62 (Treatment group); €605.82 (information group) |
| **Ghani et al. (2022)** | - QALY measured by EQ-5D-3Ld^c^  - Adjusted QoL-AD^d^, indication specific  MMSE^e^ adjusted | Direct medical costs (use of healthcare other than services provided by municipalities), from Swedish kronor to euro (2018) | Cost (€) for the participants (mean and standard error): Outpatient care cost: 6,155 (532) (IG^x^); 6,148 (510) (CG^y^) Inpatient care cost: 2,033 (417) (IG); 2,027 (385) (CG) Total cost 8,188 (762) (IG); 8,175 (751) (CG) |
| **Loohuis et al. (2022)** | -IIALY derived from the ICIQ-UI-SF^f^ symptom score, indication specific  -QALY measured by EQ-5D-5L^g^ | Direct medical and non-medical costs (medical resource use and productivity cost questionnaires (iMCQ and iPCQ)), indirect costs, in euros (2017) | Costs (IIALYs^z^): 1520 ± 3425 (IG); 1680 ± 3357 (CG); −161 (Mean difference) Costs (QALYs): 1520 ± 3425 (IG); 1680 ± 3357 (CG); −161 (Mean difference) ICER^aa^ (95% CI): € −3696 (−6716 to 12 712) ICUR^bb^ (95% CI): €6379 (−4128 to 12 769) |
| **Pelle et al. (2021)** | -QALY obtained with EQ-5D-3L, score 0.0–0.5  -QALY VAS^h^ obtained with EQ-5D-3L score 0.0–0.5  -PAM-13^i^, KOOS/HOOS^j^ | Direct medical costs (healthcare utilisation), in euros (2018) | Total health care costs during follow-up, mean +- SD^cc^ €: 439 (1,294) (IG); 496 (1,240) (CG); −31 (−66, 3) (Mean group difference (95% CI^dd^)) |
| **Röhr et al. (2021)** | -QALY, linear inter-polation of EQ-5D-5L index scores from baseline to follow-up  -PDS-5^k^, PHQ-9^l^, GAD-7^m^, PHQ-15^l^, EQ-VAS^n^, GSE^o^, SSMIS-AW^p^, SSMIS-AG^q^, SSMIS-AP^r^, SSMIS-HS^s^, LNSN, ESSI^t^, and PGI^u^ | Direct medical costs (healthcare utilisation, app maintenance), in euros (2019) | Total costs CG: Baseline: 507.95, CI: 159.21-856.69; after 4 months: 551.85, CI: 255.59-848.11; IG: Baseline: 349.46, CI: 224.10-474.82; after 4 months: 306.88, CI: 201.76-412.01 |
| **Sjöström et al. (2017)** | -QALY based on ICIQ-LUTSqol, indication-specific  ICIQ-UI SF | Costs of the RCT to determine the benefit, direct medical and non-medical costs (implementation of treatment, incontinence aids, laundry), indirect costs (time for training), from Swedish kronor in euros (2013) | Total cost: € 547.0 (IG) €482.4 (CG); App group vs control group: ∆ Cost €64.6; ∆ QALY-gain 0.00849; ICER 7615.5 |
| **Song & Kanaoka (2018)** | -QALY based on QoL^v^ scores of previous literature for dysmenorrhea, depression, or others | Direct medical and non-medical costs (application fee, utilisation of health services) and indirect costs (loss of productivity), in Japanese yen (2017) | Total cost of medical expenses (JPY 669,000 (IG); JPY 694,000 (CG)); Loss of productivity (JPY… (IG); JPY 144,000 (CG); group); Application fee (JPY 35,000 (IG); JPY(CG)) |

^a^ QALY: quality adjusted life year; ^b^ ICIQ LUTSqol: International Consultation on Incontinence Modular Questionnaire on Lower Urinary Tract Symptoms and Quality of Life; ^c^EQ-5D-3L: EuroQoL 5-Dimension 3-Level; ^d^QoL-AD: Quality of Life Alzheimer's Disease; ^e^MMSE: Mini mental State score; ^f^ICIQ-UI-SF: International Questionnaire on Incontinence - Short Form Urinary Incontinence; ^g^EQ-5D-5L: EuroQoL 5-Dimension 5-Level; ^h^VAS: Visual analog scale; ^i^PAM-13: Patient Activation Measure; ^j^KOOS/HOOS: Knee/Hip Disability Injury and Osteoarthritis Outcome; ^k^PDS-5: Posttraumatic Diagnostic Scale for DSM-5; ^l^PHQ-9/PHQ-15: Patient Health Questionnaire; ^m^GAD-7: Generalized Anxiety Disorder; ^n^EQ-VAS: EuroQoL visual analog scale; ^o^GSE: general self-efficacy; ^p^SSMIS-AW: stereotype awareness; ^q^SSMIS-AG: stereotype agreement; ^r^SSMIS-AP: stereotype application; ^s^SSMIS-HS: harm to self-esteem; ^t^ESSI: ENRICHD Social Support Inventory; ^u^PGI: Posttraumatic Growth Inventory; ^v^QoL: Quality of Life;^w^RCT: randomised controlled trial; ^x^IG: intervention group; ^y^CG: Control group; ^z^IIALY: incontinence impact adjusted life years; ^aa^ICER: incremental cost-effectiveness ratio; ^bb^ICUR: incremental cost- utility ratio; ^cc^SD: standard deviation;  ^dd^confidence interval;
