# Supplementary material for: An Overview on Methods, Evidence, and Study Quality of Health Economic Evaluation Studies for Independently Usable Digital Health Apps: Rapid Review
Source: J Med Internet Res. 2025 Aug 19;27:e68349. doi: 10.2196/68349 (PMC12364420; doi:10.2196/68349)
Supplement: Multimedia Appendix 6 [file jmir-v27-e68349-s006.docx]

### Appendix 6 – Risk of bias and methodological quality of included economic evaluations

**Consolidated Health Economic Evaluation Reporting Standards (CHEERS)**

**
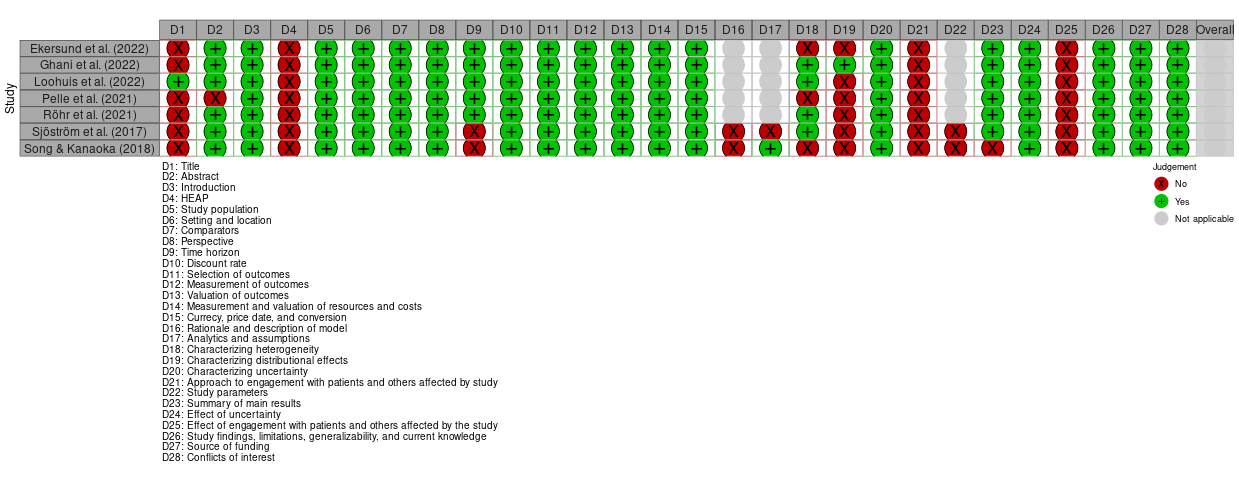
**

Figure 1: Methodological quality of full trial-based economic evaluations as judged with the CHEERS-checklist

**Consensus on Health Economic Criteria (CHEC)**


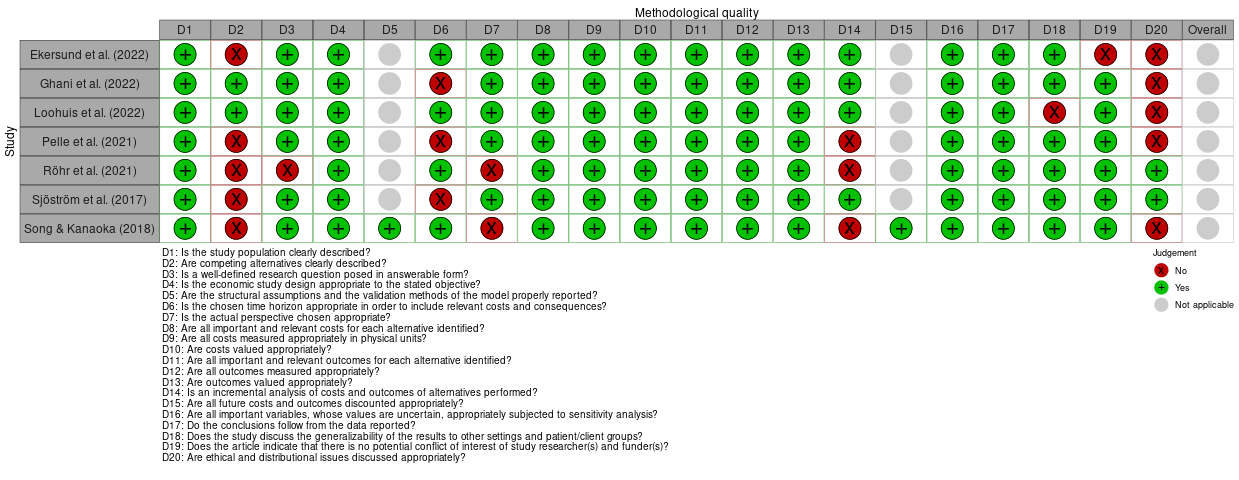


Figure 2: Methodological quality of full trial-based economic evaluations as judged with the CHEC-checklist

**Risk of Bias 2 (ROB 2)**


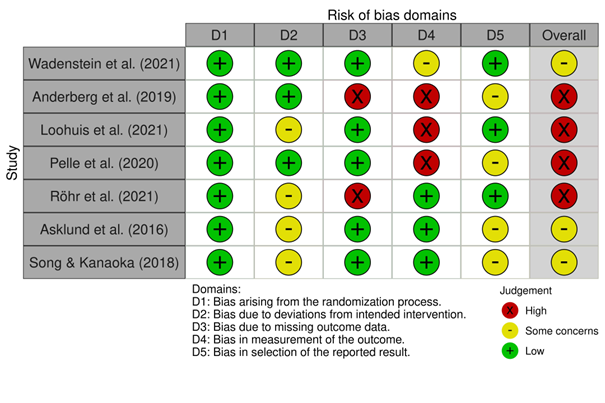
Figure 3: Risk of bias in RCT-based economic evaluations as judged with the Cochrane Risk of Bias 2 tool
